# Mismatch Negativity and P3a Impairment through Different Phases of Schizophrenia and Their Association with Real-Life Functioning

**DOI:** 10.3390/jcm10245838

**Published:** 2021-12-13

**Authors:** Giulia M. Giordano, Luigi Giuliani, Andrea Perrottelli, Paola Bucci, Giorgio Di Lorenzo, Alberto Siracusano, Francesco Brando, Pasquale Pezzella, Michele Fabrazzo, Mario Altamura, Antonello Bellomo, Giammarco Cascino, Anna Comparelli, Palmiero Monteleone, Maurizio Pompili, Silvana Galderisi, Mario Maj, The Italian Network for Research on Psychoses

**Affiliations:** 1 Department of Psychiatry, University of Campania “Luigi Vanvitelli”, 80138 Naples, Italy; luigi.giuliani.91@gmail.com (L.G.); andreaperrottelli@gmail.com (A.P.); paolabucci456@gmail.com (P.B.); brando.francesco@virgilio.it (F.B.); pezzella.pasquale3@gmail.com (P.P.); michele.fabrazzo@unicampania.it (M.F.); silvana.galderisi@gmail.com (S.G.); mario.maj@unicampania.it (M.M.); 2 Department of Systems Medicine, University of Rome Tor Vergata, 00133 Rome, Italy; di.lorenzo@med.uniroma2.it (G.D.L.); siracusano@med.uniroma2.it (A.S.); 3 Psychiatry Unit, Department of Clinical and Experimental Medicine, University of Foggia, 71122 Foggia, Italy; mario.altamura@unifg.it (M.A.); antonello.bellomo@unifg.it (A.B.); 4 Department of Medicine, Surgery and Dentistry “Scuola Medica Salernitana”, Section of Neurosciences, University of Salerno, 84133 Salerno, Italy; gcascino@unisa.it (G.C.); pmonteleone@unisa.it (P.M.); 5 Department of Neurosciences, Mental Health and Sensory Organs, S. Andrea Hospital, University of Rome “La Sapienza”, 00189 Rome, Italy; anna.comparelli@uniroma1.it (A.C.); maurizio.pompili@uniroma1.it (M.P.)

**Keywords:** schizophrenia, ERP, mismatch negativity, MMN, P3a, illness duration, real-life functioning

## Abstract

Impairment in functioning since the onset of psychosis and further deterioration over time is a key aspect of subjects with schizophrenia (SCZ). Mismatch negativity (MMN) and P3a, indices of early attention processing that are often impaired in schizophrenia, might represent optimal electrophysiological candidate biomarkers of illness progression and poor outcome. However, contrasting findings are reported about the relationships between MMN-P3a and functioning. The study aimed to investigate in SCZ the influence of illness duration on MMN-P3a and the relationship of MMN-P3a with functioning. Pitch (p) and duration (d) MMN-P3a were investigated in 117 SCZ and 61 healthy controls (HCs). SCZ were divided into four illness duration groups: ≤ 5, 6 to 13, 14 to 18, and 19 to 32 years. p-MMN and d-MMN amplitude was reduced in SCZ compared to HCs, independently from illness duration, psychopathology, and neurocognitive deficits. p-MMN reduction was associated with lower “Work skills”. The p-P3a amplitude was reduced in the SCZ group with longest illness duration compared to HCs. No relationship between P3a and functioning was found. Our results suggested that MMN amplitude reduction might represent a biomarker of poor functioning in SCZ.

## 1. Introduction

Schizophrenia is a severe mental illness with a high heterogeneity of risk factors, pathophysiology, psychopathology, and outcome [1,2,3,4,5,6,7,8,9,10,11,12,13,14,15,16,17,18,19,20,21,22,23,24,25,26,27,28,29].

People suffering from this disorder experience positive, disorganized, negative, depressive, extrapyramidal symptoms, cognitive impairment, as well as impairment in different areas of functioning [3,30,31,32,33,34,35,36,37,38,39,40,41,42,43,44,45,46,47,48]. In particular, positive symptoms usually begin with the onset of psychosis or are present in an attenuated form in the prodromal stages; they tend to recur in conjunction with the acute phases. Negative symptoms and cognitive deficits predate the onset of psychosis, might worsen when the first episode occurs, and are much more stable as compared to positive symptoms throughout the course of the illness [47,48,49].

Moreover, schizophrenia is often a chronic and relapsing disorder with incomplete symptomatic remission and variable levels of disability [50,51,52,53,54,55,56,57,58,59]. The impairment in various domains of real-life functioning, such as interpersonal relationships, everyday life skills, and work skills, represents to date the main target of care in subjects with schizophrenia since it poses a huge burden on patients, their families, and health-care systems [60,61,62,63,64,65,66,67,68,69]. It has been demonstrated that the impairment in real-life functioning is associated with different variables, some related to the illness, others to personal resources, and others to the context [66,67,68,69]. Among these variables, the duration of the illness and of untreated psychosis play a crucial role in determining a poor outcome [70,71,72,73].

The high heterogeneity in terms of pathophysiology, psychopathology, and how the illness progresses can usefully be addressed by a clinical staging approach of the illness. For this reason, the present research priorities include the identification of biomarkers of illness progression [3]. In fact, biomarkers, which are measurable indicators of biological conditions, could help to understand the pathophysiological mechanisms underpinning the poor outcome forms of the disorder, which are associated with chronic stages and high disability [74,75]. Therefore, the biomarkers can contribute to the early identification of subjects who might progress to a severe form of the illness in order to plan intensive interventions, which might control the progression of the disease and reduce the probability of poor functional outcome

Many electrophysiological indices have been used as potential biomarkers of schizophrenia. Indeed, electroencephalography (EEG) is a non-invasive, inexpensive method with a high temporal resolution that allows the identification of abnormalities of cortical brain functions and the study of the neurophysiological bases of different clinical and behavioral aspects [76,77,78,79,80,81,82,83,84,85]. Event-related potentials (ERPs) are very small brain voltages occurring in response to specific sensory, motor, or cognitive events. They have been used to investigate neurophysiological correlates of psychopathology, cognitive deficits, and functioning disturbances in subjects with schizophrenia [86,87,88,89]. In particular, the ERP components mismatch negativity (MMN) and P3 have been frequently explored in schizophrenia [90,91,92,93,94].

In the MMN-P3a auditory oddball paradigm, MMN is elicited by presenting a relatively rare deviant sound interspersed in a sequence of frequently occurring standard sounds [95], and its peak occurs generally 150–250 msec after the presentation of the stimulus, with the highest intensity recorded in temporal auditory and frontal areas [96,97,98,99,100]. In the auditory paradigm, the deviant stimulus might have a different duration (dMMN) or pitch (pMMN) with respect to the standard one [101]. MMN is an index of pre-attentive processing and sensory encoding and memory [90,102]. A reduction of MMN amplitude is frequently observed in schizophrenia [90,91,103,104]. According to a meta-analysis [92], the alterations in MMN amplitude are stable after the first years of illness throughout the life span. However, dMMN and pMMN amplitude are both reduced in subjects with chronic schizophrenia, while in the early stages of the disease, only a reduction of dMMN amplitude is present [103,104]. Therefore, the reduction of pMMN amplitude could represent an index of poor outcome and illness chronicity. Moreover, the MMN impairment has been reported also in other mental disorders, e.g., bipolar disorder, although to a lesser degree than in schizophrenia [89]. Kaur et al. showed that in subjects with first-episode psychosis, from both affective and schizophrenia spectrum, neurobiological disturbances could be already detected through reduced MMN amplitude [105]. These findings suggest that MMN alterations are linked to the psychosis dimension rather than to specific categorical diagnoses. However, MMN deficits are present during phases of clinical stability and are not associated with psychotic symptoms [106].

P3 is a positive peak that can be observed after 300 msec after the presentation of a deviant/rare stimulus during an oddball paradigm [107,108]. The P3a component is elicited by presenting rare non-target stimuli and can be observed even under passive conditions. As for the MMN, P3a might be elicited by deviant stimuli in terms of duration (dP3a) or pitch (pP3a). P3a is generated in frontal cerebral regions sustaining orientation of attention to novel stimuli. In fact, this ERP reflects early attention-mediated auditory processing, and consistent deficits of this index have been detected in subjects with schizophrenia [109,110,111,112,113] since the early stage of the disorder [90,114]. It has been demonstrated that P3 amplitude and latency are, respectively, decreased and delayed in patients with longer illness duration [110,111,115,116]. However, similar to MMN, the reduction in P3 amplitude is not specific of schizophrenia, as it can also be observed in other conditions, such as bipolar disorder and schizoaffective disorder [89,105,117].

Several studies investigated the relationship between MMN/P3a and poor outcome in subjects with schizophrenia. In particular, the impairment in MMN has been linked to cognitive and functional impairment in subjects with schizophrenia [88,90,91,118,119,120]. These relationships seem to be present since the early stages of the disorder [119,121,122,123]. On the other hand, fewer studies have examined the relationship between P3a and functioning, reporting often inconsistent results [88,90,124,125,126]. Hamilton et al. [90] investigated simultaneously the association of both MMN and P3a with functioning and assessed it using the Multidimensional Scale of Independent Functioning. Authors found that MMN but not P3a amplitude reduction was associated with the impairment in functioning of subjects with schizophrenia [90].

Although different studies investigating the relationship between ERPs and functional outcome reported associations of MMN and, to a lesser extent, of P3a with functioning measures, these results are not very robust due to some limitations. In fact, these works examined only a single or a few domain/s of functioning and did not take into account several factors that may influence real-life functioning (e.g., delusions, hallucinations, lack of insight, disorganized thinking, cognitive deficits, negative symptoms, or depression); furthermore, these studies generally included small samples of subjects with schizophrenia [88,91,118,119,120,126,127,128].

The current study aimed to investigate in clinically stable subjects with schizophrenia: (1) the impact of illness duration on MMN and P3a and (2) the relationships between MMN-P3a and real-life functioning. In order to overcome the above-reported limitations, we used the Specific Level of Functioning Scale (SLOF) for the assessment of real-life functioning. This instrument has good psychometric properties [129,130]. In contrast to other scales, it assesses multiple functional domains; providing separate scores for each domain; it can be rated on the basis of an interview with patient’s key relative/caregiver, or staff members [129,131,132,133]; and it does not include elements concerning the psychopathology or cognitive dysfunctions but evaluates the patient’s current functioning and observed behavior, focusing on person’s abilities and resources [130].

## 2. Materials and Methods

### 2.1. Study Participants

The study has been conducted as part of the add-on EEG study of the Italian Network for Research on Psychoses (Galderisi et al., 2014). One hundred and forty-eight subjects with schizophrenia (SCZ) and 70 healthy controls (HCs) were enrolled for the study at five research sites in Naples, Foggia, Rome “Tor Vergata”, Rome “Sapienza”, and Salerno. Subjects with schizophrenia were outpatients in care at the five mentioned Italian university psychiatric clinics. Inclusion criteria for patients were: a diagnosis of schizophrenia based on the DSM-IV criteria and confirmed by the Structured Clinical Interview for DSM-IV—Patient version (SCID-I-P); age between 18 and 65 years; and no treatment modifications and/or hospitalization due to symptom exacerbation in the last three months. The HCs were recruited from the community at the same research sites. The inclusion criterion for HCs was the absence of a current or lifetime Axis I or II psychiatric diagnosis.

Exclusion criteria for SCZ and HCs were: (a) a history of head trauma with loss of consciousness; (b) a history of mental retardation (moderate to severe) or of neurological diseases; (c) a history of alcohol and/or substance abuse in the last six months; (d) current pregnancy or lactation; and (e) inability to provide an informed consent.

All participants signed a written informed consent after a clear and comprehensive description of the study procedures and goals.

The electrophysiological add-on study was approved by the Ethics Committee of the involved institutions. The study has been conducted in accordance with the ethical principles of the Declaration of Helsinki.

### 2.2. Assessments

All subjects were evaluated for socio-demographic variables, such as age, education and gender, using every available source of information.

The Positive and Negative Syndrome Scale (PANSS) was administered to patients to rate positive and disorganization symptoms [134]. All items are rated on a 7-point scale from 1 (absent) to 7 (extremely severe).

The Brief Negative Symptom Scale, a second-generation rating scale [135,136], was administered to patients to assess negative symptoms according to their current conceptualization. The scale has 13 items organized into six subscales (five negative symptom subscales, Anhedonia, A-sociality, Avolition, Blunted Affect, and Alogia, and a control subscale: Distress). All the items are rated on a 7-point (0–6) scale, thus ranging from absent (0) to moderate (3) to extremely severe (6) symptoms. A total score was computed by summing the 13 individual items; subscale scores were computed by summing the individual items within each subscale [135]. Two negative symptom domains were assessed: the Experiential domain, computed by summing the scores on the subscales Anhedonia, Avolition, and A-sociality, and the Expressive deficit, calculated by summing the scores on the subscales Blunted Affect and Alogia [135].

The Calgary Depression Scale for Schizophrenia (CDSS) was used to assess depressive symptoms in SCZ [137]; the St. Hans Rating Scale (SHRS) for Extrapyramidal Syndromes assessed extrapyramidal symptoms in SCZ [138].

Neurocognitive functions were evaluated with the Measurement and Treatment Research to Improve Cognition in Schizophrenia (MATRICS) Consensus Cognitive Battery (MCCB) [139,140]. Raw scores on the MCCB were standardized to T-scores, corrected for age and gender, and based on the Italian normative sample.

Real-life functioning was assessed using the SLOF, a scale that was endorsed by the panel of experts involved in the Validation of Everyday Real-World Outcomes (VALERO) initiative as a valid measure to evaluate real-life functioning [128,141]. The SLOF is a hybrid instrument that explores many aspects of functioning, and it is based on the key caregiver’s judgment on behavior and functioning of patients. It consists of 43 items and includes the following domains: (1) physical functioning, (2) personal care skills, (3) interpersonal relationships, (4) social acceptability, (5) everyday life skills, and (6) work skills. Higher scores correspond to better functioning. In our study the SLOF was administered to the key caregiver, i.e., the person more frequently and closely in contact with the patient. For outpatients living in the community, it is possible to observe a ceiling effect for personal care skills and social acceptability; thus, according to Sabbag and colleagues [142], in our study we focused on three SLOF subscales: interpersonal relationships, everyday life skills, and work skills. The Italian version of the scale was validated as part of the Italian Network for Research on Psychoses project [129].

### 2.3. Recording Procedure

EEGs were recorded with two highly comparable EEG systems: EASYS2 (Brainscope, Prague, Czech Republic) and Galileo MIZAR-sirius (EBNeuro, Florence, Italy). In order to guarantee the same recording settings in all sites, a harmonization of the amplifier settings and recording procedures was performed. EEGs were recorded with a 29 unipolar leads cap electrode system (Fpz, Fz, Cz, Pz, Oz, F3, F4, C3, C4, FC5, FC6, P3, P4, O1, O2, Fp1, Fp2, F7, F8, T3, T4, T5, T6, AF3, AF4, PO7, PO8, Right Mastoid and Left Mastoid), placed following the 10–20 system (American Electroencephalographic Society Guidelines in Electroencephalography, 1994). All leads were referenced to earlobes (a resistor of 10 kOhm was interposed between the earlobe leads). A ground electrode was allocated on the forehead.

In order to check for artifacts, during the EEG recording, a horizontal electro-oculogram (hEOG) from the epicanthus of each eye and a vertical EOG (vEOG) from the leads beneath and above the right eye were also recorded. All impedances of the leads were kept below 5 kΩ. The EEG data were filtered with a band-pass of 0.15–70 Hz. The sampling rate was 512 Hz. Before each session, a calibration was carried out for all channels with a 50 μV sine wave.

MMN and P3a were recorded through a stereo headset during the presentation of 2400 tones (80 db SPL), of which 83.3% were standard tones (50 msec, 1000 Hz), 8.3% duration (d) deviant tones (100 msec, 1000 Hz), and 8.3% pitch (p) deviant tones (50 msec, 1200 Hz), with an interstimulus interval of 450 msec. During stimuli presentation subjects were asked to watch a silent animated cartoon, and after the paradigm ended, they were asked some questions regarding the video (test duration = 20 min).

For each recording, subjects were invited to relax and to minimize movements or muscle tension.

Participants were invited not to drink coffee or tea and abstain from smoking cigarettes in the 2 h before the recording session and not to take the psychotropic drugs during the morning. If the subject reported a non-restoring sleep during the night prior to the recording, EEG session was postponed.

### 2.4. EEG Data Analysis

The pre-processing analyses were performed by one expert from the coordinating center (Naples) using Brain Vision Analyzer software (Brain Products, Munich, Germany). In order to characterize MMN and P3a deflections, data were parsed into epochs of 1000-mc duration, which were time-locked to the onset of the cue and spanned from a 100-mc pre-stimulus period up to 900 msec post-stimulus. The recorded EEG was digitally filtered offline using a band-pass filter of 1–30 Hz. MMN and P3a waves were extracted in each subject by the averaging method on all the “deviant” trials separately for duration and pitch deviant trials in order to ameliorate the signal/noise ratio, ruling out baseline activity not related to the stimulus. Trials with drifts larger than ±75 μV in any scalp electrode were refused. If, following artifacts and noisy trials removal, less than 100 usable trials for either duration or deviant trials (50% of d- or p-deviant trials) remained, the subject was excluded from the analysis. Data were baseline-corrected using the 100-msec time window preceding stimuli. For MMN analysis, peaks resulting from the presentation of standard tones, duration deviant (dMMN), and pitch deviant (pMMN) were automatically marked using the “peak finder” function of Brain Analyzer, with the most negative point ranging from 90–250 msec. Then, we subtracted the standard tone waveform from the duration deviant and pitch deviant ones. For both subtraction waves (pMMN and dMMN), the amplitude was then measured. P3a peaks were automatically marked using the “peak finder” function of Brain Analyzer, with the most positive point ranging from 230–380 msec after pitch (pP3a) and duration deviant (dP3a) stimuli. According to previous literature, MMN peak was analyzed from Fz and P3a from Cz [119,143].

### 2.5. Statistical Analyses

SPSS Version 22.0 (IBM Corporation, 2014; Armonk, NY, USA) was used to perform all statistical analyses.

SCZ were divided into four groups using quartiles of the illness duration.

Pearson’s χ^2^ test was performed to evaluate differences on gender distribution between groups.

Analyses of variance (ANOVA) and covariance (ANCOVA) were used to test group differences on continuous variables. Bonferroni post-hoc comparisons were conducted following significant ANOVA F-tests.

Spearman’s rank correlations were performed to test the relationships between MMN and P3a with real-life functioning domains. Furthermore, if correlations were statistically significant, we performed partial correlations to exclude the influence of possible confounding factors (positive, negative, disorganized, depressive, and extrapyramidal symptoms as well as cognitive impairment).

## 3. Results

### 3.1. Subject Characteristics

One hundred and forty-eight SCZ and 70 HCs were originally enrolled in the study. However, 23 SCZ and four HCs did not complete the paradigm for MMN-P3a recording. Furthermore, eight SCZ and five HCs were excluded for the presence of many artifacts in the ERP recordings. Thus, 117 SCZ and 61 HCs were included in the present analysis.

Data on relevant demographic and clinical characteristics of the study sample are provided in Table 1.

Gender distribution was significantly different between the two groups (χ^2^ = 6.42; *p* = 0.01) since, in the SCZ group, the number of male subjects was higher as compared to HCs. There was no significant difference in the mean age between the two sample groups (F = 2.257; *p* = 0.135). Furthermore, as expected, SCZ had significantly lower education as compared to controls (F = 7.139; *p* = 0.008). SCZ had a mild severity of both positive and disorganization symptoms (PANSS mean dimension score < 9 for both dimensions) and mild to moderate severity of the negative symptoms (BNSS total score of 34.70 ± 16.381). Finally, SCZ showed low scores of depression (CDSS total score < 4) and of parkinsonism (SHRS Parkinsonism score < 1) (Table 1).

Based on quartiles of illness duration, SCZ were divided into four groups: SCZ-A, ≤ years (*n* = 23); SCZ-B, 6 to 13 years (*n* = 38); SCZ-C, 14 to 18 years (*n* = 27), and SCZ-D > 18 years (19 to 32 years, *n* = 29). Table 2 shows demographic and clinical details of the four patients’ groups. Subjects with the longest illness duration (SCZ-D) had a significantly higher positive symptom score than patients with the shortest illness duration (SCZ-A) (*p* = 0.008). Furthermore, SCZ-D group had a significantly higher global parkinsonism score (SHRS) and lower cognitive skills (MCCB) compared to the SCZ-A (respectively, *p* = 0.008; *p* = 0.015) and SCZ-B groups (respectively, *p* = 0.003; *p* = 0.033).

### 3.2. Group Differences on ERPs

Group comparisons for the amplitude of MMN and P3a, elicited by duration (dMMN, dP3a) and pitch (pMMN, pP3a) deviants, were made between the five sample groups (HCs, SCZ-A, SCZ-B, SCZ-C, and SCZ-D), controlling for age and gender.

There was a significant group effect on dMMN (F = 8.3, *p* < 0.001) and pMMN (F = 7.5, *p* < 0.001) amplitudes. Post-hoc pairwise comparisons demonstrated that all groups of SCZ, compared to HCs, showed reduced dMMN (all *p* < 0.001) and pMMN amplitudes (SCZ-A< HCs, *p* = 0.01; SCZ-B < HCs, *p* = 0.03; SCZ-C and SCZ-D< HCs, *p* < 0.001), while no statistically significant difference was observed between patients’ groups.

**Figure 1:**
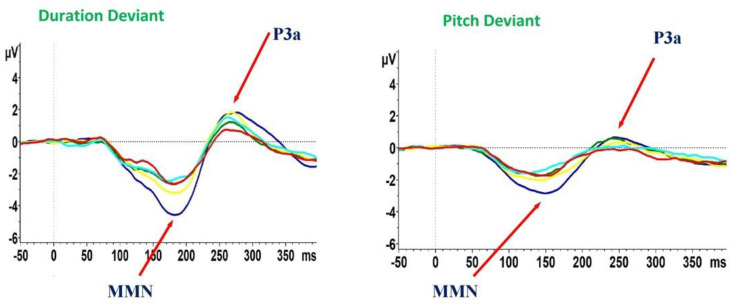
Mismatch negativity (MMN) and P3a waveforms recorded during the auditory paradigm in healthy controls and subjects with schizophrenia. HCs, healthy controls (blue line); SCZ, subjects with schizophrenia; ID, illness duration. SCZ-A, ID ≤ 5 (green line); SCZ-B, ID 6 to 13 years (yellow line); SCZ-C, ID 14 to 18 years (pale blue line); SCZ-D, ID 19 to 32 years (red line).

In addition, there was a group effect on dP3a (F = 2.5, *p* = 0.04); however, this result did not survive the correction for multiple tests. Follow-up post-hoc pairwise comparisons demonstrated that this effect was driven by differences between SCZ-D and HCs (SCZ-D < HCs, *p* = 0.003), while no differences were found between patients’ groups. In addition, we did not find any significant difference between the five groups for pP3a amplitude (F = 2.1, *p* = 0.078) (Figure 1, Table 3).

Furthermore, since MMN and P3a amplitudes could be influenced by different factors, we also performed control analyses in order to reveal the possible effect of confounding factors on these results. In particular, we performed analysis of covariance in order to evaluate differences between the four groups of patients on MMN-P3a parameters, controlling for age, gender, positive symptoms, neurocognition, and global parkinsonism.

We did not find any statistically significant difference in the MMN and P3a amplitude (*p* > 0.05) among the four SCZ groups as well as when we controlled for the possible effects of the confounding variables.

### 3.3. Correlation Analyses

**Figure 2:**
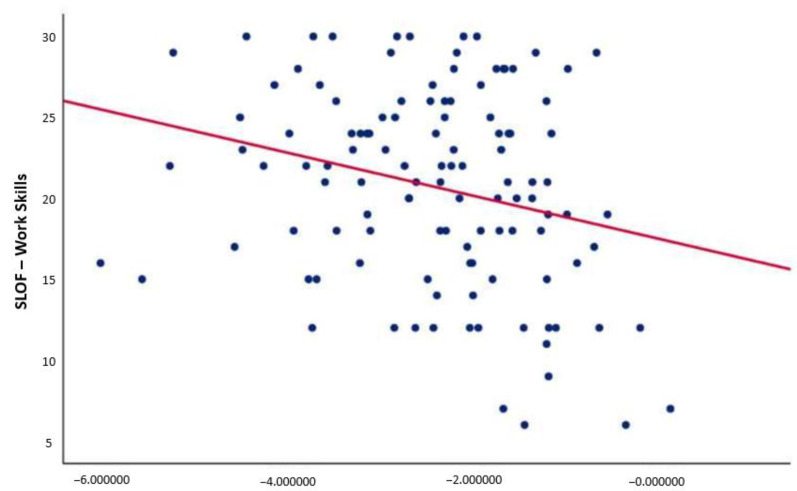
Correlation between p-MMN amplitude and the “work skills” domain of the SLOF scale. p-MMN, pitch deviant mismatch negativity. Negative correlation between p-MMN amplitude and the “work skills” domain of the SLOF scale (r = −0.257; *p* = 0.005) (significant *p*-value threshold 0.008). This correlation remained significant after controlling for positive, negative, and disorganized symptoms; depression; neurocognition; and global parkinsonism.

Correlation analyses revealed a negative relationship between pMMN amplitude and the “work skills” domain of the SLOF scale (r = −0.257; *p* = 0.005) (Figure 2). This correlation remained significant after controlling for positive, negative, and disorganized symptoms; depression; neurocognition; and global parkinsonism. No correlation was found between P3a and real-life functioning in SCZ.

### 3.4. Additional Analyses

Additional control analyses were performed to test differences in MMN and P3a between between two subgroups of subjects with schizophrenia, divided on the basis of the “work skills” domain scores. We reported methods and results of this analysis within the Supplementary materials and Supplementary Tables S1–S3.

## 4. Discussion

The main results of our study included: (1) a reduction of MMN amplitude for pitch and duration deviant stimuli in all groups of subjects with schizophrenia as compared to healthy controls, independently from illness duration, age, gender, positive symptoms, neurocognition, and global parkinsonism; (2) subjects with a longer duration of illness had reduced dP3a amplitude as compared to healthy controls; and (3) in SCZs, pMMN was correlated with the “work skills” domain of the SLOF.

In line with previous findings, MMN amplitude was reduced in subjects with chronic schizophrenia compared to healthy controls, for both pitch and duration deviant stimuli [103,104]. Furthermore, as expected, our results showed that MMN was reduced independently from illness duration and other factors, such as age, gender, positive symptoms, neurocognition, and parkinsonism. Different studies reported that the impairment in MMN amplitude is present since the early stages of the illness as well as in subjects with chronic schizophrenia [90,91,103,104]. This MMN amplitude impairment is stable after the first years of illness, and it is not progressive throughout the life span [92]. Our results suggested that subjects with schizophrenia do present deficits in pre-attentive processing, as indexed by reduced MMN amplitude and that these deficits are independent from illness progression. Thus, deficit in MMN might represent a possible stable trait marker of schizophrenia, allowing early diagnosis and hopefully early intervention in subjects with schizophrenia.

With respect to P3a, we found that there was a weak group effect on dP3a amplitude; however, this result did not survive the correction for multiple tests. In particular, this effect was driven by the fact that patients with longer illness duration showed reduction in dP3a amplitude as compared to healthy controls, while no significant difference was found between patient’s groups. Although we did not find any difference in pP3a and dP3a across different stages of the disease, our study showed a trend of P3a amplitude reduction in the group with the highest illness duration. These results are in line with previous studies which demonstrated that P3 amplitude is reduced in patients with longer illness duration [110,111,115,116]. Therefore, these findings suggest that P3, reflecting early attention-mediated auditory processing, might represent a marker of illness progression. However, whether P3a represent a marker of schizophrenia progression has to be further investigated, and more studies are needed to confirm this finding. Indeed, results of previous studies on the topic are controversial: some of these studies reported that P3a amplitude is reduced mainly in patients with longer illness duration, while some others found this alteration also in first-episode psychosis patients and at-risk subjects [90,110,111,113,114,116]. Moreover, some studies demonstrated that P3a amplitude is affected by antipsychotic administration [144], suggesting that the progressive P3a amplitude reduction across illness stages might depend on the use of antipsychotic drugs instead of the illness progression. Our results cannot add to this debate, as we could not compare drug-treated and untreated subjects.

As regard to the relationship of MMN and P3 with measures of functioning, according to a previous study [90], we found that only MMN and not P3a amplitude negatively correlated with real-life functioning in subjects with schizophrenia. In particular, we found an association between MMN amplitude reduction and impairment in the “work skills” domain of the SLOF. This finding of association between MMN and functioning was also supported by the additional analyses performed testing the differences between two subgroups of subjects with schizophrenia divided on the basis of the “work skills” domain scores.

Previous works reported in subjects with schizophrenia a relationship of MMN amplitude deficit with functional impairment and psychosocial and socio-occupational disability [88,90,91,118,119,120]. This association has been identified since the early stages of the disease [119,121,122,123]. On the contrary, scarce and inconsistent findings have been reported about the association between P3a amplitude and functioning [88,90,124,125,126].

As said before, MMN is an index of basic cognitive processes, which are usually impaired in subjects with schizophrenia [46,47,48,49]. Our results concerning the association between MMN and functioning might be interpreted in the light of the influence of deficits in cognitive processes on functioning in subjects with schizophrenia, a finding which has been extensively reported in literature [49,145,146]. This relationship is complex and mostly indirect, with many variables, such as social cognition, negative symptoms, and functional capacity, acting as mediators and moderators in the pathway from cognitive impairment to functioning [66,67,68,69,145,147]. Moreover, cognitive deficits are associated with everyday life skills, independent living, and occupational functioning [145]. This is in line with our results, which provide a deeper knowledge about the impact of basic cognitive processes alterations, as indexed by MMN amplitude reduction, on functioning in subjects with schizophrenia. In the light of these observations, further studies are encouraged in order to evaluate the pathways towards functioning impairment starting from pre-attentive processing deficits.

The strengths of our study stem from the fact that it overcomes different limitations of previous studies investigating associations between ERPs and functioning. As a matter of fact, previous studies on the topic examined only a single or fewer domain/s of functioning; they did not take into account symptoms and cognitive deficits that may affect real-life functioning; they collected only information from patients that could be influenced by many factors (e.g., delusions, hallucinations, lack of insight, disorganized thinking, cognitive deficits, negative symptoms, or depression); and they had usually small samples [88,91,118,119,120,126,127,128]. In order to overcome these limitations, we used a large sample of stabilized subjects with schizophrenia, and we assessed the functioning through the SLOF, which assesses multiple functional domains, and the scoring is based on patient’s key relative/caregiver or staff members. Furthermore, this instrument does not include elements concerning psychopathology or cognitive impairment but evaluates the patient’s current functioning and observed behavior, focusing on person’s abilities and resources.

As a limit of the present study, the possible confounding effect of the pharmacological treatment should be taken into account, as we could not control for the dosage of the antipsychotic medications. However, subjects with schizophrenia with a longer illness duration (from 19 to 32 years) had a significantly higher global parkinsonism score (which might be regarded as an indirect measure of the use and dosage of antipsychotics) as compared to subjects with illness duration ≤ 5 or from six and 13 years. Therefore, in order to test the possible effect of confounding factors on group comparison for MMN-P3a, we used as covariates the global parkinsonism score alongside with other variables that were different across the SCZ subgroups (age, gender, positive symptoms, and neurocognition), with no change in the results. In addition, with regard to correlation analyses between MMN-P3a measures and functioning, we also performed partial correlations, controlling for variables that might affect the results, such as global parkinsonism; positive, negative, and disorganized symptoms; depression; and neurocognition, with no change in the results.

However, for a clear interpretation of our findings, further studies, including drug-naïve subjects at their first episode as well as subjects at high risk for psychosis, are needed to confirm that MMN reduction is an index of poor functional outcome.

In conclusion, our results demonstrated that deficits in pre-attentive processing, as indexed by MMN reduction, are key aspects of schizophrenia. In fact, these deficits have been reported already in the prodromal stage of the disorder, remain stable through the lifespan, and are associated with poor real-life functioning. Therefore, MMN amplitude reduction might represent a possible stable trait biomarker of schizophrenia, and thus, it might help clinicians in predicting the functional outcome and implementing early and effective treatment strategies for patients with these deficits.

## Figures and Tables

**Table 1:** Demographic and clinical characteristics of the study sample.

Demographic and Clinical Information	HC (*n* = 61)	SCZ (*n* = 117)	F/χ₂	*p*
Gender (M/F)	31/30	82/35	6.420	**0.01**
Age (years, mean ± SD)	33.8 ± 12.276	36.25 ±9.116	2.257	0.135
Education (years, mean ± SD)	13.95 ± 4.084	12.51 ± 2.999	7.139	**0.008**
Paternal Education (years, mean ± SD)	10.43 ± 4.612	9.97 ± 4.91	0.344	0.559
Maternal Education (years, mean ± SD)	9.818 ± 4.41	9.183 ± 4.0556	0.844	0.360
BNSS Total score (mean ± SD)		34.70 ± 16.381		
BNSS Expressive deficit domain (mean ± SD)		11.30 ± 7.31		
BNSS Experiential domain (mean ± SD)		21.10 ± 9.185		
PANSS Positive (mean ± SD)		8.32 ± 4.727		
PANSS Negative (mean ± SD)		15.65 ± 5.843		
PANSS Disorganization (mean ± SD)		8.64 ± 3.604		
CDSS Total score (mean ± SD)		3.23 ± 3.835		
SHRS global parkinsonism (mean ± SD)		0.86 ± 1.149		
SLOF Interpersonal relationships (mean ± SD)		23.09 ± 5.725		
SLOF Everyday life skills (mean ± SD)		46.85 ± 6.834		
SLOF Work Skills (mean ± SD)		20.72 ± 6.10		
MCCB Neurocognitive Composite Score (mean ± SD)		35.18 ± 10.902		
Duration of illness (mean ± SD)		12.98 ± 8.067		
Type of AP medication (%)		78.4% second-generation AP 11.2% first-generation AP 10.3% both AP		

AP, antipsychotic; BNSS, Brief Negative Symptom Scale; CDSS, Calgary Depression Scale for Schizophrenia; HCs, Healthy controls; MCCB, MATRICS Consensus Cognitive Battery; PANSS, Positive and Negative Syndrome Scale; SCZ, subjects with schizophrenia; SD, standard deviation; SHRS, The St. Hans Rating Scale for extrapyramidal syndromes; SLOF, The Specific Level of Functioning scale. *p* values in bold indicate statistical significance.

**Table 2:** Demographic and clinical characteristics of the four patients’ groups, composed by subjects with different illness duration (SCZ-A, ID ≤ 5; SCZ-B, ID 6 to 13 years; SCZ-C, ID 14 to 18 years; SCZ-D, ID 19 to 32 years).

Demographic and Clinical Information	SCZ-A (23)	SCZ-B (38)	SCZ-C (27)	SCZ-D (29)	F/χ₂	*p*
Age (years, mean ± SD)	26.87 ± 6.75	33.1 ± 6.031	37.41 ± 4.925	46.62 ± 6.34	50.82	<0.001 *
Gender (M/F)	19/4	22/16	19/8	22/7	4.877	0.181
Education (years, mean ± SD)	11.87 ± 2.68	12.76 ± 3.16	12.70 ± 3.074	12.52 ± 3.03	0.471	0.703
Paternal Education (years, mean ± SD)	9.65 ± 4.380	10.89 ± 4.9	10.42 ± 4.851	8.29 ± 5.238	1.51	0.217
Maternal Education (years, mean ± SD)	10.20 ± 3.75	9.368 ± 3.91	9.923 ± 4.3811	7.32 ± 3.761	2.604	0.056
BNSS Tot (mean ± SD)	30.13 ± 18.5	34.26 ± 15.3	36.58 ± 14.409	37.29 ± 17.7	0.951	0.419
Expressive deficit (mean ± SD)	10.57 ± 7.80	10.21 ± 6.99	11.92 ± 6.603	12.79 ± 7.99	0.803	0.495
Experiential domain (mean ± SD)	18.22 ± 10.8	21.74 ± 8.51	21.73 ± 7.754	22.04 ± 9.83	0.953	0.418
PANSS Positive (mean ± SD)	5.83 ± 2.552	8.24 ± 4.037	8.77 ± 4.616	10.07 ± 6.19	3.751	0.013 **
PANSS Negative (mean ± SD)	14.74 ± 6.69	16.47 ± 5.72	14.54 ± 4.35	16.32 ± 6.49	0.872	0.458
PANSS Disorganization (mean ± SD)	7.35 ± 2.145	8.50 ± 3.790	9.04 ± 3.504	9.54 ± 4.194	1.72	0.167
CDSS Tot (mean ± SD)	2.78 ± 4.552	3.61 ± 3.803	2.96 ± 3.538	3.36 ± 3.654	0.273	0.845
SHRS global parkinsonism (mean ± SD)	0.52 ± 0.846	0.55 ± 0.86	0.89 ± 1.05	1.54 ± 1.503	5.35	0.002 ***
SLOF Interpersonal relationships (mean ± SD)	23.43 ± 5.73	22.97 ± 6.21	3.30 ± 4.681	22.75 ± 6.22	0.076	0.973
SLOF Everyday life Skills (mean ± SD)	48.17 ± 6.7	47.34 ± 5.72	46.30 ± 6.638	45.64 ± 8.451	0.698	0.555
SLOF Work Skills (mean ± SD)	23.04 ± 5.62	21.32 ± 5.82	19.52 ± 5.905	19.14 ± 6.609	2.28	0.083
Neurocognitive Composite Score (mean ± SD)	38.57 ± 9.28	36.97 ± 11.5	35.93 ± 10.321	29.43 ± 10.29	3.99	0.010 ****

BNSS, Brief Negative Symptom Scale; CDSS, The Calgary Depression Scale for Schizophrenia; HCs, Healthy controls; MCCB, MATRICS Consensus Cognitive Battery; PANSS, Positive and Negative Syndrome Scale; SCZ, subjects with schizophrenia; SD, standard deviation; SHRS, The St. Hans Rating Scale for extrapyramidal syndrome; SLOF, The Specific Level of Functioning scale. *p* values in bold indicate statistical significance. Post-hoc pairwise comparisons: * For age, each group differs from the others (all *p* < 0.001); ** SCZ-D had higher PANSS positive score compared to SCZ-A (*p* = 0.008); *** SCZ-D had higher SHRS global parkinsonism score compared to SCZ-A (*p* = 0.008) and SCZ-B (*p* = 0.003); **** SCZ-D had lower cognitive performance compared to SCZ-A (*p* = 0.015) and SCZ-B (*p* = 0.033).

**Table 3:** Group differences for MMN and P3a. Age and gender as covariates.

MMN-P3a Amplitude	HCs (*n* = 61)	SCZ-A (*n* = 23)	SCZ-B (*n* = 38)	SCZ-C (*n* = 27)	SCZ-D (*n* = 29)	F	*p*
d-MMN	−5.51 ± 2.47	−3.456 ± 1.83	−3.87 ± 2.05	−3.55 ± 1.71	−3.208 ± 1.99	8.274	<0.001 *
p-MMN	−3.50 ± 1.56	−2.43 ± 1.129	−2.70 ± 1.29	−2.11 ± 0.930	−2.35 ± −1.19	7.533	<0.001 *
d-P3a	2.95 ± 1.95	2.02 ± 1.81	2.54 ± 1.79	2.13 ± 1.11	1.53 ± 1.21	2.5	0.04 **
p-P3a	1.52 ± 1.05	1.40 ± 1.26	1 ± 1.16	0.95 ± 0.87	0.70 ± 1.01	2.1	0.078

HCs, healthy controls; SCZ, subjects with schizophrenia; d-MMN, duration deviant MMN; p-MMN, pitch deviant MMN; d-P3a: duration deviant P3a; p-P3a, pitch deviant P3a. *p* values in bold indicate statistical significance (significant *p*-value threshold 0.002). Post-hoc pairwise comparisons: * All SCZ groups had reduced d-MMN (all *p* < 0.001) and p-MMN (SCZ-A< HCs, *p* = 0.01; SCZ-B < HCs, *p* = 0.03; SCZ-C and SCZ-D< HCs, *p* < 0.001) amplitude compared to HCs; ** SCZ-D had reduced d-P3a amplitude compared to HCs (*p* = 0.003).

## Data Availability

All data supporting the findings of this study are available within the article and Supplementary materials.

## Supplementary Materials

The following are available online at https://www.mdpi.com/article/10.3390/jcm10245838/s1, Additional control analyses, Methods and Results. Table S1: Demographic and clinical characteristics of the two patient subgroups; Table S2: Group differences for MMN and P3a amplitudes and Table S3: Group differences for MMN and P3a amplitudes (positive and disorganized symptoms and global parkinsonism as covariates).
