# Supplementary material for: Mismatch Negativity and P3a Impairment through Different Phases of Schizophrenia and Their Association with Real-Life Functioning
Source: J Clin Med. 2021 Dec 13;10(24):5838. doi: 10.3390/jcm10245838 (PMC8707866; doi:10.3390/jcm10245838)
Supplement: Supplementary file 1 [file jcm-10-05838-s001.zip › jcm-1500407-supplementary.pdf]

Journal of
Clinical Medicine

Supplementary materials

Additional control analyses

Methods

Additional control analyses were performed to test differences in MMN and P3a between two subgroups of subjects with schizophrenia (SCZ) with high and low scores on the "work skills" domain of the Specific Levels of Functioning (SLOF) scale. We used a median split analysis on the scores of the SLOF "work skills" domain, to classify SCZ as patients with low work skill scores, SCZ-LW (SLOF < 21), and as patients with high work skill scores, SCZ-HW (SLOF ≥ 21). Pearson's χ^2 test was performed to evaluate differences on gender distribution between groups. Analyses of variance (ANOVA) and covariance (ANCOVA) were used to test group differences on continuous variables, and in particular on MMN and P3a amplitude. Positive symptoms, disorganization and global parkinsonism were used as covariates, to control for main confounding factors.

Results

Table S1 provides demographic and clinical characteristics of the two groups. As expected, SCZ-LW had more severe positive ($p = 0.001$), disorganized ($p = 0.034$) and extrapyramidal ($p = 0.032$) symptoms, as compared to SCZ-HW (Table S1).

Table S1. Demographic and clinical characteristics of the two patient subgroups.

Demographic and clinical information	SCZ-HW (n = 64)	SCZ-LW (n = 53)	F	p
Age (years, mean ± SD)	36.27 ± 9.37	36.12 ± 8.93	0.008	0.930
Gender (M/F)	45/19	36/17	0.16	0.900
Education	12.42 ± 3.24	12.62 ± 2.74	0.118	0.732
Paternal Education (years, mean ± SD)	9.53 ± 4.63	10.47 ± 5.28	0.732	0.332
Maternal Education (years, mean ± SD)	8.85 ± 4.04	9.42 ± 3.93	0.537	0.465
BNSS Tot (mean ± SD)	32.30 ± 15.41	37.60 ± 17.19	3.029	0.085
PANSS Positive (mean ± SD)	7.03 ± 3.62	9.88 ± 5.43	11.316	**0.001**
PANSS Negative (mean ± SD)	14.43 ± 6.04	17.13 ± 5.28	6.399	**0.013**
PANSS Disorganization (mean ± SD)	8.00 ± 3.60	9.42 ± 3.49	4.582	**0.034**
CDSS Tot (mean ± SD)	2.79 ± 3.28	3.77 ± 4.39	1.857	0.176
SHRS global parkinsonism (mean ± SD)	0.66 ± 1.13	1.12 ± 1.13	4.731	**0.032**
Neurocognitive Composite Score (mean ± SD)	36.38 ± 12.02	33.94 ± 9.35	1.390	0.241
SLOF Work Skills (mean ± SD)	25.28 ± 2.95	15.10 ± 3.85	260.3	**0.0001**

BNSS: Brief Negative Symptom Scale; CDSS: The Calgary Depression Scale for Schizophrenia; MCCB: MATRICS Consensus Cognitive Battery; PANSS: Positive and Negative Syndrome Scale; SD: Standard Deviation; SHRS: The St. Hans Rating Scale for extrapyramidal syndrome; SCZ: subjects with schizophrenia; SCZ-HW: subjects with schizophrenia with high work skills domain scores; SCZ-LW: subjects with schizophrenia with low work skills domain scores; SLOF: The Specific Level of Functioning scale. p values in bold indicate statistical significance.

There was a significant group effect for dMMN amplitude ($F = 5.85$, $p = 0.017$) and for pMMN ($F = 6.51$, $p = 0.012$), while we did not find any significant difference between the two groups for dP3a ($F = 0.048$, $p = 0.827$) and pP3a ($F = 1.74$, $p = 0.189$) amplitude (Table S2). Controlling for variables that were different across the two sample subgroups (positive and disorganized symptoms and global parkinsonism), we found that there was a significant group effect for pMMN amplitude ($F = 5.76$, $p = 0.018$), while we did not find any significant difference between the two groups for dMMN ($F = 3.82$, $p = 0.053$), dP3a ($F = 0.299$, $p = 0.586$) and pP3a amplitude ($F = 1.38$, $p = 0.242$) (Table S3).

Table S2. Group differences for MMN and P3a amplitudes.

MMN-P3a amplitude	SCZ-HW (n = 64)	SCZ-LW (n = 53)	F	p
d-MMN	−3.93 ± 1.71	−3.08 ± 2.08	5.85	**0.017**
p-MMN	−2.66 ± 1.04	−2.12 ± 1.25	6.51	**0.012**
d-P3a	2.07 ± 1.58	2.13 ± 1.55	0.48	0.827
p-P3a	1.12 ± 1.12	0.86 ± 1.07	1.74	0.189

d-MMN: duration deviant MMN; d-P3a: duration deviant P3a; p-MMN: pitch deviant MMN; p-P3a: pitch deviant P3a; SCZ: subjects with schizophrenia; SCZ-HW: subjects with schizophrenia with high work skills domain scores; SCZ-LW: subjects with schizophrenia with low work skills domain scores. *p* values in bold indicate statistical significance.

Table S3. Group differences for MMN and P3a amplitudes (positive and disorganized symptoms and global parkinsonism as covariates).

MMN-P3a amplitude	SCZ-HW (n = 64)	SCZ-LW (n = 53)	F	p
d-MMN	−3.93 ± 1.71	−3.08 ± 2.08	3.82	0.053
p-MMN	−2.66 ± 1.04	−2.12 ± 1.25	5.76	**0.018**
d-P3a	2.07 ± 1.58	2.13 ± 1.55	0.299	0.586
p-P3a	1.12 ± 1.12	0.86 ± 1.07	1.38	0.242

positive and disorganized symptoms and global parkinsonism as covariates. d-MMN: duration deviant MMN; d-P3a: duration deviant P3a; p-MMN: pitch deviant MMN; p-P3a: pitch deviant P3a; SCZ: subjects with schizophrenia; SCZ-HW: subjects with schizophrenia with high work skills domain scores; SCZ-LW: subjects with schizophrenia with low work skills domain scores. *p* values in bold indicate statistical significance.

These findings confirmed the main outcome of the study, that is the association of MMN and not P3a with real life functioning in subjects with schizophrenia, controlling for other possible confounders.
